# Therapeutic potential of propranolol in central nervous system disorders

**DOI:** 10.3389/fpsyt.2026.1752025

**Published:** 2026-08-17

**Authors:** Jia-Ning Zhao, Hui-Ying Zhang, Jing-Yu Yang, Yong-Yu Yin, Li-Ming Zhang

**Affiliations:** 1 School of Life Sciences and Biopharmaceuticals, Shenyang Pharmaceutical University, Shenyang, China; 2 Academy of Military Medical Sciences, Beijing, China

**Keywords:** anxiety, CNS, prop, PTSD, β-blocker

## Abstract

**Introduction:**

Propranolol (Prop), a classic non-selective β-adrenergic receptor antagonist, has long played a significant role in the treatment of cardiovascular diseases such as hypertension and arrhythmias. Compared with its peripheral regulatory effects, the potential therapeutic value of Prop in neurological disorders such as post-traumatic stress disorder (PTSD), memory dysfunction, autism spectrum disorder (ASD), anxiety, migraine prophylaxis, and essential tremor is gradually being re-evaluated.

**Methods:**

This review aims to integrate the latest evidence from clinical and preclinical studies, systematically reviewing the effects of Prop on neurological diseases and its potential mechanisms.

**Results:**

Specifically, we explore its translational value from the perspective of β-adrenergic receptor subtype-specific regulation, thereby assessing its potential for precision medicine in neurology.

**Discussion:**

Finally, this review outlines novel therapeutic strategies targeting β receptors for treating central nervous system (CNS) diseases, with the ultimate goal of providing a robust theoretical foundation and clear translational pathways for future drug development.

## 1 Introduction

Central nervous system (CNS) diseases refer to a series of conditions affecting the structure or function of the brain or spinal cord, leading to neurological or psychiatric complications (1). These conditions range from mild neurological dysfunctions that may affect motor, sensory, visual, speech, cognitive performance, or combinations thereof, to states of coma and brain death (2). Damage to or specific degeneration of different types of neurons within the CNS can lead to neurological diseases such as Alzheimer’s disease (AD), Parkinson’s disease (PD), ischemic stroke, and spinal cord injury (1). With the aging of the global population, the number of people living with CNS diseases has increased dramatically, with approximately one-sixth of the global population affected, imposing a substantial economic burden on society (3). Despite years of research, many CNS diseases remain clinical challenges due to their high incidence among middle-aged and older adults, complex pathogenesis, long treatment cycles, and limited diagnostic and therapeutic options (4).

Currently, clinical treatment primarily employs receptor modulators and neurotransmitter inhibitors. Within this therapeutic backdrop, β-blockers have garnered significant attention for their applications in CNS diseases. Propranolol (Prop), a representative β-blocker (5), exhibits high lipophilicity and favorable blood-brain barrier permeability, enabling it to exert regulatory effects within the CNS. Studies have demonstrated that Prop can modulate the release of neurotransmitters such as norepinephrine and serotonin by blocking β-adrenergic receptors, thereby influencing neuronal excitability and synaptic plasticity. In recent years, the therapeutic potential of Prop in CNS disorders—including post-traumatic stress disorder, anxiety disorders, and migraine—has been progressively elucidated, offering novel directions for its clinical application.

Prop is widely used to treat cardiovascular diseases such as hypertension, arrhythmias, and angina pectoris. The molecular formula of Prop is C_16_H_21_NO_2_, with a molecular weight of 259.34 g/mol (6). Its chemical structure consists of a naphthalene ring (hydrophobic group) and a propanolamine side chain (containing an isopropylamine group), and it contains a chiral center with two optical isomers (S- and R-forms). The unique chemical structure of Prop confers high lipophilicity, allowing it to readily cross the blood-brain barrier (BBB) and exert specific regulatory effects within the CNS. During emotional excitement or exercise, sympathetic nerve activation leads to an increased heart rate and enhanced myocardial contractility, which can be effectively alleviated by the β-adrenergic blocker Prop (7). Prop likely achieves its blood pressure-lowering effect through multiple mechanisms, including reducing cardiac output and peripheral vascular resistance (8). The therapeutic applications of Prop have expanded beyond the cardiovascular system, with its effects on the ocular system and the CNS being well documented (9). Its mechanism of action in treating tremors may be related to the blockade of β2 receptors in the CNS, thereby reducing tremors caused by sympathetic excitation, such as essential tremor. Furthermore, the central effects of Prop can also lead to some adverse reactions, such as drowsiness and dizziness (10). Prop primarily exerts its physiological regulatory effects by targeting adrenergic β1, β2, and β3 receptors (see **Table 1**).

**Table 1:** The distribution and function of β receptors in the body.

Classification	Distribution	Function	References
β1 receptor	Heart, brain, juxtaglomerular (JG) cells, and adipose tissue	Enhances myocardial contractility; accelerates heart rate and conduction velocity; regulates the function of the HPA axis and promotes the expression of neurotrophic factors; promotes renin release and controls renin secretion; and promotes lipolysis.	(11–14)
β2 receptor	Bronchial smooth muscle, vascular smooth muscle, gastrointestinal smooth muscle, skeletal muscle, liver, and mast cells	Promotes bronchial smooth muscle relaxation; promotes vascular smooth muscle relaxation; promotes gastrointestinal smooth muscle relaxation; inhibits the reconsolidation of emotional memories; and regulates the activity of the amygdala.	(11, 13, 15, 16)
β3 receptor	Adipose tissue, brainstem, hypothalamus, and other regions	Activation leads to an increase in mitochondrial biogenesis and activity in white adipose tissue and briefly increases insulin release. Promotes the browning of white adipose tissue and reduces neuroinflammation.	(17, 18)

**Figure 1:**
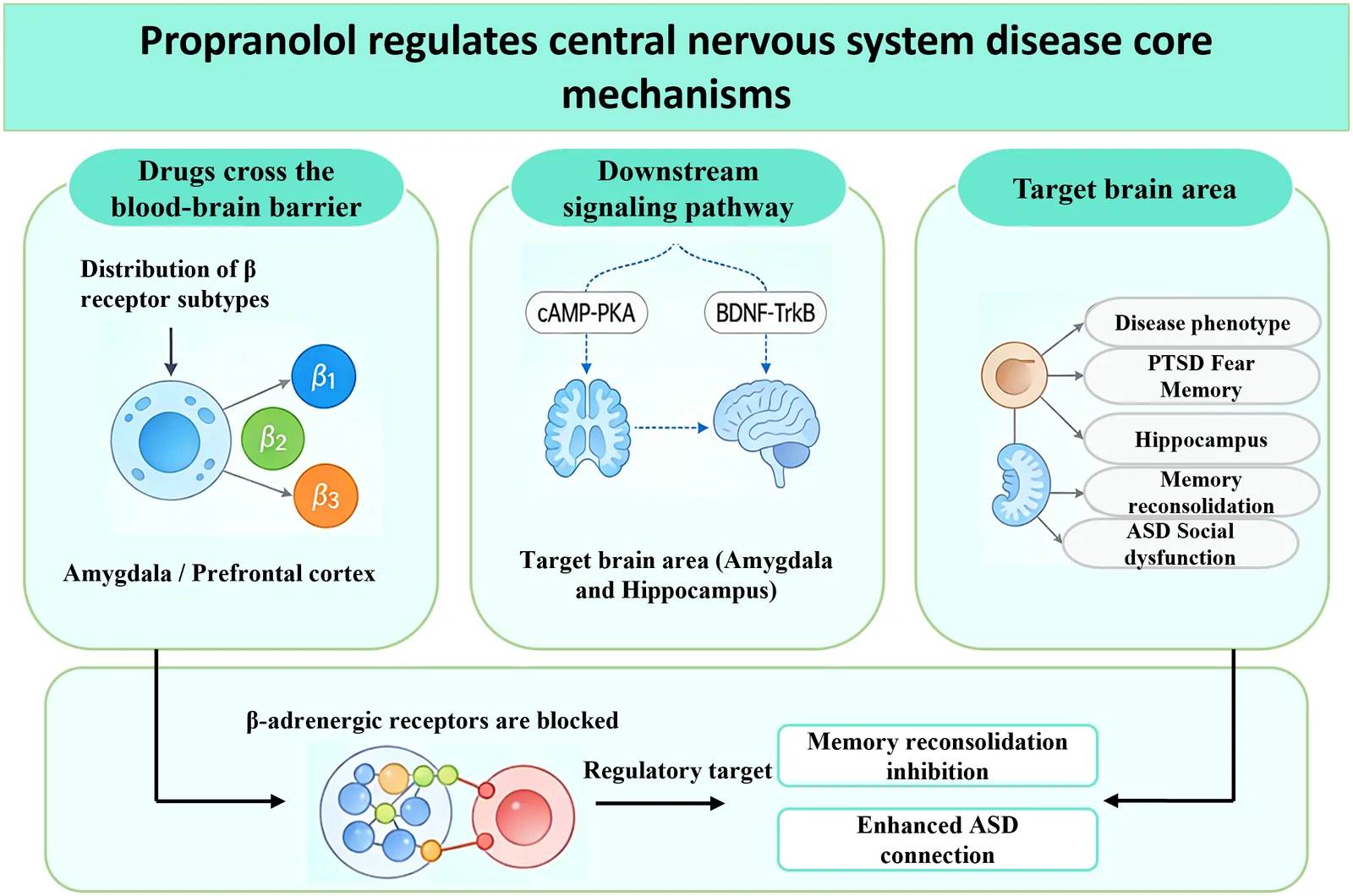
Overview of the evidence for the actions of Prop in different CNS diseases.

Notably, given these clinical challenges, the therapeutic potential of Prop in specific CNS disorders such as post-traumatic stress disorder (PTSD) and anxiety disorders has become a prominent focus of clinical research. Building upon this interest, this review systematically examines the current evidence regarding the effects of Prop on core CNS functions and its emerging therapeutic actions in related diseases. It aims not only to provide a comprehensive perspective on Prop’s multifaceted actions in CNS pathophysiology, but also to critically assess the existing evidence and lay a robust foundation for its future clinical translation in neuropsychiatric disorders. To summarize the actions of Prop in various neurological disorders such as PTSD, anxiety, depression, autism spectrum disorder (ASD), and cognitive dysfunction (CD), this review presents a schematic diagram integrating and visually summarizing the available evidence and potential application areas (see **Figure 1**).

## 2 Methods

This review considers Prop as the core research subject, with a focus on investigating its therapeutic potential in CNS disorders. The literature search was conducted based on the PubMed database, and the inclusion criteria were limited to English-language clinical studies and review abstracts published from 1971 to 2025. The keywords adopted in the search strategy included: “Prop” or “Central Nervous System” or “β-blocker” or “Post-Traumatic Stress Disorder” AND “Anxiety” or “Cognitive Dysfunction” or “Autism Spectrum Disorder” or “Parkinson’s Disease” or “Depression” or “Anxiety”. It should be specifically noted that all literature for which full texts could not be obtained through legitimate channels was excluded from the scope of the final analysis.

## 3 Post-traumatic stress disorder

Post-traumatic stress disorder (PTSD) is a complex psychiatric condition that can develop after exposure to a traumatic event (e.g., terrorist attack, physical violence, unexpected death of a loved one) and is characterized by long-term enhancement of fear memory and impaired extinction (19, 20). Patients with PTSD face severe psychological distress due to intrusive flashback memories and repeated trauma re-experiencing, which severely impairs their physical and mental health (21). According to the memory reconsolidation theory (22), under certain conditions, memory retrieval can lead to memory destabilization and subsequent restabilization (reconsolidation), a process that can be disrupted pharmacologically (23). Prop is the most studied drug in clinical research on memory reconsolidation (see **Table 2**). Studies have revealed that Prop can selectively interfere with the consolidation process of emotional memories formed under different stress conditions. Prop crosses the BBB and potentially prevents the protein synthesis required for the reconsolidation of such emotional memories, thereby producing a lasting degradation of the memory trace (24). Furthermore, Prop can interfere with memories formed under single stress conditions, but its interference effect on emotional memories under combined stress conditions depends on the timing of administration. This finding suggests that Prop interferes with memory consolidation by acting on specific neural pathways, which are activated to varying degrees under different stress conditions.

**Table 2:** Summary of key findings on PTSD and Prop.

Research topic	Core discovery	References
PTSD pathologies	Long-term fear memory is enhanced, and extinction is impaired after trauma. Recurrent flashbacks seriously damage physical and mental health.	(19, 20).
Memory reconsolidation theory	The extracted memory can enter the “destabilization and restabilization” process, which can be modulated by pharmacological interventions.	(22)
Central mechanism of action	Prop crosses the blood-brain barrier and inhibits the synthesis of proteins required for emotional memory reconsolidation.	(24).
Randomized double-blind trial	Activation of traumatic memory combined with Prop significantly reduced CAPS and PCL-S scores.	(25)
Fear generalization	Prop can block the enhancement of memory reconsolidation caused by norepinephrine and reduce fear generalization.	(26)
Latest meta-analysis	Seven studies on clinical symptoms and three studies on physiological indicators showed that the effects of Prop on PTSD symptoms require further research.	(27)
Clinical conclusion	The available evidence is insufficient to support Prop’s routine use for PTSD memory intervention.	(27)

Prop demonstrates unique advantages in modulating fear memory generalization. Some studies indicate that Prop disrupts the reconsolidation of memories enhanced by noradrenergic stimulation and prevents fear generalization (26). A randomized double-blind trial found that Prop reduced physiological response levels in patients with post-traumatic stress disorder compared to placebo (25). This suggests that Prop, combined with reactivation of traumatic memory, could significantly alleviate PTSD symptoms as measured by the Clinician-Administered PTSD Scale (CAPS) and the patient-rated PTSD Checklist-Specific (PCL-S) (28). However, other studies have failed to replicate these findings. For example, another randomized controlled trial indicated that Prop did not disrupt fear responses in healthy female volunteers upon re-exposure to threatening stimuli. (29). To determine the efficacy of Prop in disrupting traumatic memory, reducing symptoms, and modulating physiological responses in patients with PTSD, Raut et al. (27) conducted a meta-analysis that screened 3,224 publications and ultimately including seven studies evaluating the effects of Prop on PTSD symptoms and three studies evaluating its effects on physiological responses. The meta-analysis found no significant beneficial effect of Prop on PTSD symptoms. Collectively, current evidence is insufficient to support the effectiveness of Prop in disrupting traumatic memories in patients with PTSD; therefore, its routine clinical use for this purpose warrants further investigation.

## 4 Cognitive dysfunction

Cognitive dysfunction (CD) refers to a clinical syndrome involving a persistent decline in one or more cognitive domains, including attention (30), learning and memory, language, executive function, visuospatial ability, and social cognition (31). Studies have found that approximately 10%–15% of individuals over the age of 65 develop CD, with more than half progressing to dementia within five years (32). CD has been identified in PTSD and is associated with impaired traumatic memory processing, increased severity of psychiatric symptoms, and functional impairment. Previous studies have shown that Prop is one of the most commonly used substances in humans to block the consolidation and reconsolidation of memory (33). Psychological stress, such as public speaking, affects the performance of healthy individuals on tasks that require cognitive flexibility, and this effect can be reversed by the antagonistic effect of β-adrenergic. This finding supports the hypothesis that cognitive flexibility disorders related to stress are associated with the norepinephrine system. Prop is a synthetic β-adrenergic receptor blocker that crosses the BBB, exerting peripheral effects on the noradrenergic system and central inhibitory effects on protein synthesis, which is necessary for the consolidation of new learning into long-term memory storage (34). Animal studies have shown that the infusion of protein synthesis inhibitors into the amygdala within a limited consolidation window leads to memory impairment in subsequent fear-conditioning tasks. Protein synthesis is also required for memory reconsolidation; post-retrieval infusion of protein synthesis inhibitors results in impaired long-term memory while leaving short-term memory intact. Prop is one of several protein synthesis inhibitors used in animal studies to dampen the salience of emotional memories.

Additionally, a randomized, double-blind, placebo-controlled trial assessed changes in cognitive function using the subtests of the Wechsler Adult Intelligence Scale-III (WAIS-III) in patients with chronic PTSD following up to one week of Prop treatment. The study found that PTSD patients treated with Prop (1 mg/kg) performed significantly better on a composite measure of processing speed, suggesting that Prop administration may improve cognitive function in patients with PTSD (35). A recent study published in Biological Psychiatry revealed that although administering Prop prior to fear memory retrieval significantly weakened the memory retrieval process, the drug failed to promote fear extinction learning and may instead have inhibited it. (36). This finding aligns with controversial results in the existing literature regarding Prop’s effects on extinction learning. Research suggests that the timing of Prop administration may have bidirectional effects (37). Administration after memory recall may support extinction by interfering with reconsolidation, while pre-retrieval administration (especially at least one day after learning) may lead to enhanced fear responses later in the absence of the drug. Prop, which is commonly used to treat hypertension, arrhythmias, angina, and acute anxiety, has been experimentally shown to reverse stress-related cognitive deficits (38).

One study found that aging is associated with worsening cognitive abilities and an increased risk of neurodegenerative diseases (39). Hypertension is the most prevalent modifiable risk factor for global cardiovascular morbidity and mortality, and clinical data indicate that it is also a risk factor for Alzheimer’s disease (AD). Prop can improve cognitive impairment and regulate AD-related markers displayed by senescence-accelerated mouse prone-8 (SAMP8) mice. Administration of Prop (5 mg/kg for 3 weeks) to 6-month-old SAMP8 mice alleviated the cognitive memory impairment these mice showed in novel object recognition tests (39).

## 5 Anxiety

Anxiety is a multidimensional emotional state characterized by excessive worry about future uncertainty or potential threats, manifesting as persistent tension, fear, and unease, accompanied by autonomic activation (e.g., increased heart rate, sweating, and muscle tension) and impaired cognitive function (40). Many mental disorders are comorbid with anxiety (41), and they have significant psychological, social, and economic impacts. Currently available anxiolytic drugs (such as barbiturates and benzodiazepines) have major central effects and little peripheral impact, consistent with evidence that anxiety depends on the CNS, particularly the activity of the limbic system (amygdala, hypothalamus, and associated connecting structures) (42). Early studies showed that Prop could alleviate symptoms such as palpitations caused by anxiety by inhibiting sympathetic excitation within the CNS. This mechanism of action makes it clinically useful for treating anxiety-related symptoms. The action of Prop within the CNS is relatively complex, and its specific mechanisms are still under investigation. A meta-analysis on panic disorder showed that Prop was comparable to benzodiazepines for short-term treatment, but evidence supporting long-term efficacy is lacking (43). Furthermore, one study reported that Prop’s improvement of subjective anxiety is limited, possibly because of its primary action on peripheral rather than central β-receptors (44). Prop is commonly used to treat migraines, tachycardia, and performance anxiety. Due to its effects on the noradrenergic system, it is also indicated as a second-line therapy for anxiety disorders. The use of Prop to mask the physical symptoms of anxiety is well established. This drug can reduce the physiological symptoms of anxiety, including changes in blood pressure, heart rate, respiratory rate, and skin conductance. According to a crossover study (45), patients with anxiety disorders were assessed as significantly improved after two weeks of Prop treatment compared with two weeks of placebo. Patients with chronic anxiety received Prop at a dose of 160 mg daily. After two weeks of treatment, Prop markedly reduced the physical manifestations of anxiety and significantly relieved other symptoms related to β-stimulation, including shortness of breath, chest pain, and weakness (46, 47). Experimental results indicate that taking Prop immediately after retrieving an emotional memory can lead to post-reactivation amnesia, i.e., a weakening of the subsequent expression of the memory. Prop has been shown to have acute effects on fear-potentiated startle responses. Furthermore, research on Prop suggests it can alleviate stress in less severe situations, such as test anxiety, stage fright, performance anxiety in musicians, and fear of surgery (48).

Fear is a key behavioral component of both PTSD and anxiety disorders (49). Exposure therapy based on fear extinction is a crucial component of psychotherapy for anxiety disorders and PTSD. Attempts have been made to combine exposure therapy with drugs targeting fear memory retrieval and reconsolidation to enhance treatment efficacy. The noradrenergic (NA) signaling system has received particular attention due to its role in regulating stress responses and its involvement in fear and learning processes. Research has found that Prop can alter the activity of the polymorphic layer, composed of neurons, mossy cells, and GABAergic interneurons, significantly reducing memory trace reactivation in the dorsal dentate gyrus (dDG) (50). A case report described worsening depressive episodes in three patients following Prop treatment for medical conditions, with a dose-dependent effect and rapid relief upon discontinuation of Prop (43). However, studies of animal phobias suggested that Prop does not produce significant effects, indicating that its efficacy may vary depending on the type of fear. Numerous clinical studies indicate the considerable potential of Prop for treating anxiety disorders. However, preclinical research on the anxiolytic activity of Prop has rarely been reported, posing significant challenges to investigating its mechanisms of action. Future studies should conduct additional preclinical evaluations and mechanistic investigations of Prop’s anxiolytic effects, comprehensively analyzing potential regulatory mechanisms from the perspectives of effector brain regions, neural circuits, and cellular and molecular levels to lay an important research foundation for developing next-generation anxiolytic drugs based on β2-adrenergic receptors.

## 6 Depression

Depression is one of the most prevalent and debilitating mental disorders, posing a major public health challenge worldwide (51). It is projected that by 2030, depression will become the most commonly diagnosed disease globally. The etiology of depression is complex, and because the number of studies investigating the association between Prop and depression is limited, the relationship between the two remains controversial. Some researchers have conducted tests on anxiety-like and depression-like behaviors, social interaction, and learning and memory functions. For example, at the preclinical level, one study found that both Prop and nadolol protected Sprague-Dawley rats from displaying anxiety-like or depression-like behaviors. (52).

It was noted that bisoprolol and Prop did not induce depressive symptoms, unlike metoprolol, which, in most studies, showed a positive effect on the development or worsening of depressive symptoms. Based on an analysis of medical data, the authors concluded that β-blockers have no significant effect on the induction of depressive symptoms or that this effect is clinically insignificant (53). They also suggested that selected groups of these drugs may prevent the development of depression or reduce its symptoms. Moreover, a positive effect of β-blockers on reducing the level of anxiety was noted (54). Furthermore, they described a 42-year-old woman who developed severe depression, marked apathy, social withdrawal, and anorexia after three months of treatment with Prop (80 mg daily) for hypertension; when the dose was reduced to 40 mg daily, her depressive symptoms significantly improved within five days and completely resolved within eight days (55). Existing clinical observational data have several methodological limitations. First, the majority of case reports lack a baseline assessment of patients’ pre-treatment emotional state, making it difficult to exclude the possibility that depressive symptoms existed before medication use. Second, the target populations themselves have a high baseline incidence of depression, a confounding factor that substantially impacts causal inference (56). Given these research limitations, elucidating the exact mechanism by which Prop may contribute to the development of depression remains a highly challenging scientific problem. Future research should adopt multicenter, prospective study designs combined with multidimensional data, such as genomics and neuroimaging, to systematically examine the temporal relationship and dose–response relationship between drug exposure and depressive symptoms.

## 7 Autism spectrum disorder

Autism spectrum disorder (ASD) is characterized by early-onset deficits in social communication and repetitive sensorimotor behaviors (57). Individuals with ASD frequently experience co-occurring anxiety and depressive symptoms (58), which can further exacerbate social functional impairment. In recent years, Prop has shown promising therapeutic potential for addressing the multidimensional symptoms of ASD. For instance, recent clinical double-blind studies revealed that 12 weeks of Prop treatment significantly improved anxiety symptoms in patients with ASD (59, 60), underscoring its utility in managing these comorbid conditions. Importantly, Prop appears to exert beneficial effects on improving verbal fluency in patients with ASD. For instance, one study reported that Prop significantly improved the performance of patients with ASD in dialogue interaction tasks and their scores in non-verbal domains. (60). Notably, the significant potential of Prop to improve cognitive function in patients with ASD has garnered attention. For example, **s**cientists found that acute administration of Prop significantly improved the performance of patients with ASD on an anagram task, suggesting its potential therapeutic value for enhancing cognitive abilities in ASD (57). Future studies should expand the sample size to further explore Prop’s efficacy in this aspect. Another study found impaired working memory in patients with ASD, and a single dose of Prop intervention can significantly improve working memory performance (61). Extensive neuroimaging studies have shown abnormal functional connectivity between brain regions in patients with ASD (62, 63). Recent clinical research suggests that functional connectivity between brain regions may be related to the cognitive benefits of Prop in ASD. For example, Prop was found to modulate functional connectivity within the default mode network in patients with ASD, specifically by significantly increasing brain functional connectivity during task execution (60). This change in functional connectivity may partly explain Prop’s effect on improving cognitive function in ASD. In addition, multiple MRI studies have further demonstrated that the cognitive benefits of Prop in patients with ASD may be closely related to the modulation of functional connectivity between brain regions in ASD (64, 65). Patients with ASD exhibit abnormal functional connectivity and impaired cognitive performance, and Prop treatment may alleviate abnormal functional connectivity in key brain regions such as the frontopolar cortex (FPC) in patients with ASD (64).

In summary, although the application of Prop in treating multidimensional symptoms of ASD remains investigational, its potential benefits for language, cognition, and mood are increasingly evident (66). Future studies should expand sample sizes, evaluate long-term efficacy and safety of Prop, and develop precision treatment strategies to optimize therapeutic outcomes for individuals with ASD.

## 8 Parkinson’s disease

Parkinson’s disease (PD) is a neurodegenerative disorder characterized primarily by the loss of dopaminergic neurons in the CNS, which leads to classic motor symptoms such as resting tremor, bradykinesia, rigidity, and postural instability, alongside various non-motor manifestations (67). Notably, PD ranks as the second most common neurodegenerative disease globally (68). Furthermore, over the past decade, the prevalence and incidence of PD have risen by more than 30%, and conservative projections indicate that more than 12 million individuals globally will be affected by PD by 2050. Regarding its treatment, PD is commonly treated with levodopa (69) or Madopar as monotherapy (70), but their efficacy is often suboptimal. In this context, an early clinical trial explored a combination therapy of Prop and levodopa in patients with PD (71). Notably, this combination had a significant additive therapeutic effect on tremor compared with the effect of levodopa alone. Tremor improved in 13 out of 18 patients treated with both levodopa and Prop, whereas improvement was observed in only 6 out of 18 patients treated with levodopa alone (72). It is important to note that Prop is a primary β-blocker used to treat tremor, which could itself be an early sign of PD. Paradoxically, a body of epidemiological evidence has raised questions about its role in disease risk. In three studies (73–75), there was an inverse association (decreasing in magnitude) between the duration of β‐antagonist use and the risk of PD (76, 77). In two studies (78, 79), Prop remained significantly associated with an increased risk of PD after 1–2 years and 2–8 years of use, with estimates decreasing in both studies with longer durations of use. Two studies reported no association with increasing duration of use of β2‐agonists (80, 81). For instance, compared with never using β-blockers, ever use of β-blockers was associated with a 41% increased risk of PD. Moreover, pooled effect estimates for different β-antagonists yielded varying results, with Prop itself being associated with a 2.36-fold increased risk of PD. This has prompted research into whether β-blockers or β-agonists influence the risk of developing PD. Nevertheless, the majority of studies in this area have significant limitations. In the case of Prop, a key methodological issue is “confounding by indication”, since it is often prescribed to treat essential tremor, a condition that can be misdiagnosed as early PD (82).

To systematically present the research evidence regarding Prop in different CNS disorders, the following table summarizes the available evidence (see **Table 3**), focusing on six core diseases: PTSD, CD, anxiety, depression, ASD, and PD. It summarizes evidence from three dimensions: core evidence types, evidence level strength, and key research support directions. The table clarifies differences in the completeness and reliability of the drug’s evidence chain across diseases, from preclinical mechanism exploration to clinical application verification, providing an intuitive reference for accurately evaluating its therapeutic potential and identifying future research directions.

**Table 3:** Levels of evidence across CNS disorders.

Disorder	Evidence type	Level of evidence	Key research findings
PTSD	Large-scale meta-analyses, multicenter RCTs, and preclinical mechanistic studies	High level, with a relatively complete evidence chain, but some controversies	β-receptor-mediated inhibition of fear memory reconsolidation; improvement of CAPS/PCL-S scores by combined trauma memory activation and medication; regulation of fear generalization
CD	Preclinical animal models, small-scale RCTs, retrospective studies	Moderate level, with limited clinical evidence but clear preclinical mechanisms	Improvement of trauma-related cognitive flexibility impairments; reversal of cognitive and memory deficits in SAMP8 mice; modulation of neural activity in the amygdala and hippocampus
Anxiety	Large-scale meta-analyses, medium-sample RCTs	High level with sufficient evidence for short-term efficacy	Alleviation of anxiety-related physiological symptoms (abnormal heart rate, blood pressure, etc.); comparable short-term efficacy to benzodiazepines; improvement of specific situational anxiety (e.g., performance anxiety, dental phobia)
Depression	Case reports, preclinical animal experiments, and small-scale observational studies	Low level, with conflicting evidence and limited clinical support	Some studies show improvement in depression-like behaviors; some case reports indicate potential induction of depressive symptoms, regulation of HPA axis function, and inhibition of neuroinflammation
ASD	Small-scale RCTs, case series, and preclinical mechanistic studies	Moderate level with accumulating clinical evidence	Improvement in social communication deficits and comorbid anxiety; modulation of functional connectivity within the default mode network; enhancement of working memory and verbal fluency
PD	Medium-sized RCTs, controlled clinical studies, and meta-analysis	High level, with clear efficacy for tremor control but a controversial association with disease risk	Additive therapeutic effect on tremor when combined with levodopa; regulation of motor circuits through β-receptor blockade; controversial association with PD risk (confounded by indication bias)

## 9 Potential risks of long-term use of Prop

Prop has served as a cornerstone therapy for hypertension and cardiovascular disorders for decades. Nevertheless, its prolonged administration warrants careful consideration of potential adverse effects, which clinicians and patients should weigh against its therapeutic benefits. The risk profile of chronic Prop therapy is contingent upon two primary factors: the administered dose and the underlying neurological condition being treated. Consequently, distinct CNS disorders exhibit markedly heterogeneous risk patterns (54, 59). In PTSD, sustained high-dose Prop administration has been associated with attenuated emotional responsiveness, a phenomenon attributed to the drug’s dampening of amygdala-mediated emotional memory consolidation. Importantly, these effects appear to be reversible upon dose reduction or treatment cessation. Paradoxically, parallel evidence suggests that Prop may confer cognitive benefits in this population, specifically enhancing information processing speed (28, 83).

Conversely, patients with pre-existing cognitive impairment may experience compromised learning and memory function with prolonged use, attributable to hippocampal beta-adrenergic receptor blockade (84). The lipophilic nature of Prop facilitates blood-brain barrier penetration, increasing susceptibility to CNS side effects, though this risk is considerably attenuated at lower doses (39). In anxiety disorders, chronic high-dose administration commonly produces physical fatigue and attentional deficits through sympathetic nervous system suppression, whereas intermittent low-dose strategies appear to mitigate these concerns. It is worth noting that Prop primarily addresses somatic anxiety manifestations (tachycardia and tremor) rather than subjective psychological distress (43).

Regarding depression, the relationship is nuanced: while isolated reports associate high-dose use with depressive symptomatology, recent longitudinal data indicate a minimal risk of inducing depression, with some studies even suggesting protective effects. Low-dose protocols rarely precipitate mood disturbances, and any emergent symptoms generally resolve with dose adjustment (54). Notably, in autism spectrum disorder, extended low-dose Prop therapy has not demonstrated emotional blunting or cognitive deterioration in controlled trials; rather, significant anxiety reduction has been observed with favorable tolerability profiles (59, 85). Similarly, patients with Parkinson’s disease receiving low-dose Prop adjunctively with levodopa report primarily peripheral effects (bradycardia and asthenia) without evidence of emotional or cognitive compromise, while benefiting from meaningful tremor reduction through modulation of stress hormone-mediated motor cortex hyperactivity (86).

## 10 Limitations

This review has several limitations. First, as a narrative review without a pre-registered systematic methodology (e.g., PRISMA), our evidence collection may have inadvertently omitted some relevant studies, limiting the comprehensiveness and strength of our conclusions. Future reviews should employ standardized strategies, including dual screening and risk-of-bias assessment, to enhance reliability. Second, this review lacks a detailed discussion of long-term risks (e.g., emotional blunting and cognitive changes) associated with Prop use in CNS disorders, largely due to the scarcity of long-term prospective studies. This gap underscores the urgent need for systematic safety evaluations across diverse populations. Third, the available research on depression remains extremely limited, precluding a comprehensive analysis comparable to that conducted for other disorders covered in this review. This paucity of evidence significantly restricts our ability to draw meaningful conclusions regarding Prop’s therapeutic potential in depression. Fourth, although we highlight controversial findings in PTSD, depression, and PD, our analysis remains insufficient. Future reviews should systematically dissect these methodological factors to clarify sources of discrepancy. Future research should prioritize prospective, long-term studies with standardized outcomes to validate Prop’s therapeutic potential and inform personalized treatment strategies.

## 11 Conclusions

As demonstrated in this review, Prop is used clinically primarily to treat hypertension and migraine, and to decrease heart rate. The application of Prop in treating various types of anxiety or stress, including stage fright and PTSD, has a well-documented molecular basis. Due to its high lipophilicity and ability to cross the BBB, Prop exerts effects on the CNS in addition to its peripheral actions.

As a pleiotropic drug, Prop can appropriately be termed a multi-target agent. Therefore, it can be used to treat complex disorders that manifest with both psychiatric and somatic effects simultaneously. In recent years, continuous in-depth research on the pathogenesis of various diseases and drug targets has been conducted. Future studies will not only focus on the potential use of Prop as an adjunct to cognitive therapy for PTSD but will also explore its applications in other disease areas. Future research should focus on improving the understanding of its mechanisms of action and developing individualized treatment plans to enhance the safety and effectiveness of its clinical application.

In the neuropsychiatric field, research on Prop is extending from its traditional adjunctive treatment role for anxiety and PTSD to a wider range of CNS disorders. While the prospects are broad, the CNS effects of Prop remain controversial. For example, does long-term use lead to emotional blunting or changes in cognitive function? How can efficacy and safety be balanced through dose optimization in different populations (e.g., the elderly and children)? These questions urgently require more rigorous clinical research. In the future, personalized treatment strategies incorporating genomics and biomarker testing may improve the precision of Prop therapy. Concurrently, the development of novel β-blockers could further refine pharmacological profiles and mitigate adverse effects. Having evolved from an antihypertensive agent to a versatile therapeutic tool, Prop’s “crossover” trajectory continues to unfold.

In summary, research on the application of Prop in CNS disorders is continually advancing. While its established indications in migraine prophylaxis and essential tremor remain the cornerstone of its neurological use, its potential in alleviating emotional disturbances and treating emerging CNS disorders related to neuroinflammation deserves further exploration. Although primarily indicated for cardiovascular conditions, its potential for alleviating emotional disturbances and treating CNS disorders associated with neuroinflammation deserves further exploration. Future studies may reveal additional insights into the mechanisms and applications of Prop in the treatment of CNS disorders. Overall, Prop may be beneficial for age-related brain dysfunction and could emerge as a candidate drug for treating other neurodegenerative diseases.
